# Biochemical Approaches on Commercial Strains of *Agaricus subrufescens* Growing under Two Environmental Cultivation Conditions

**DOI:** 10.3390/jof8060616

**Published:** 2022-06-09

**Authors:** Daiana Almeida, Rossana V. C. Cardoso, Carla Pereira, Maria José Alves, Isabel C. F. R. Ferreira, Diego Cunha Zied, Wagner G. Vieira Junior, Cinthia E. C. Caitano, Ângela Fernandes, Lillian Barros

**Affiliations:** 1 Centro de Investigação de Montanha (CIMO), Instituto Politécnico de Bragança, Campus de Santa Apolónia, 5300-253 Bragança, Portugal; daiana@ipb.pt (D.A.); rossana@ipb.pt (R.V.C.C.); carlap@ipb.pt (C.P.); maria.alves@ipb.pt (M.J.A.); iferreira@ipb.pt (I.C.F.R.F.); 2 AquaValor—Centro de Valorização e Transferência de Tecnologia da Água—Associação, Rua Dr. Júlio Martins N° 1, 5400-342 Chaves, Portugal; 3 Faculdade de Ciências Agrárias e Tecnológicas (FCAT), Campus Dracena, Universidade Estadual Paulista, São Paulo 17900-000, Brazil; dczied@gmail.com; 4 Programa de Pós-Graduação em Microbiologia Agropecuária, Faculdade de Ciências Agrárias e Veterinárias (FCAV), Universidade Estadual Paulista (UNESP), São Paulo 14884-900, Brazil; vieira.jr@unesp.br (W.G.V.J.); cinthia.cardoso@unesp.br (C.E.C.C.)

**Keywords:** *Agaricus subrufescens*, cultivation system, nutritional value, chemical composition, bioactive properties

## Abstract

In the present work, the effect of the cultivation process, in the field and under a controlled environment, on biochemical parameters by using commercial strains of *A. subrufescens* were evaluated. The results obtained revealed that the strains cultivated in the field presented higher levels for most of the parameters evaluated (organic acids (20.5–48.0 g/100 g dw), tocopherols (107.0–198.6 µg/100 g dw), and phenolic acids and related compounds (245.2–359.0 µg/100 g dw and 10.6–23.7 µg/100 g dw, respectively)), except for the carbohydrates (53.4–72.6 g/100 g dw), energetic value (373–380 Kcal/100 g dw), and total free sugars (28.8–43.1 g/100 g dw), parameters in which the strains grown in a controlled environment present better results. For both cultivation systems, similar results were obtained regarding saturated, monounsaturated, and polyunsaturated fatty acids, as well as antioxidant and antimicrobial activities. These data contribute to the knowledge and highlight the characterized strains and the cultivation process, which can be used to obtain ingredients with potential applicability as a source of functional compounds.

## 1. Introduction

Global mushroom production has grown considerably, representing an increase of 90%, with the genera *Agaricus*, *Lentinula*, *Pleurotus*, *Auricularia*, and *Flammulina* being the five most produced [1]. According to estimates provided by FAOSTAT (Food and Agriculture Organization), in 2019 China was the main producer (9,672,406 tons), followed by Japan (508,572 tons), the United States (415,471 tons), Poland (392,141 tons) and Holland (324,620 tons) [2].

*Agaricus subrufescens* Peck. is also known as *Agaricus blazei* Murrill and *Agaricus brasiliensis* Wasser, but from a nomenclatural point of view, several taxonomists proposed and agreed with the unique denomination of *A. subrufescens*. This species has common names such as “sun mushroom” in Brazil, “himematsutake” in Japan, and “almond mushroom” in the USA, due to its benzaldehyde and benzoic acid content, which gives it a unique almond aroma and flavor, and make it a particularly popular gourmet food [3,4,5].

This mushroom is used as a therapeutic food, as it prevents diseases such as hypertension, cancer, diabetes, hyperlipidemia, chronic hepatitis and is known to positively impact the immune system [6,7]. These properties are related to the presence of bio-molecules such as phenolic compounds, fatty acids, ergosterol, carotenoids, vitamins, terpenes. These compounds have been reported to be responsible for the antioxidant, antimicrobial, anti-tumor, and other bioactive properties of *A. subrufescens* [8,9,10]. With the discovery of its biochemical properties and consequently its value in the international market, the production of *A. subrufescens* grew in countries such as Japan, China, Brazil, and the United States, which are the biggest producers of this mushroom [11,12,13]. Cultivation is a critical phase in the mushroom production process and significantly influences its biochemical composition.

The main environmental factors encompass intrinsic factors, such as composition of substrates, sources of nitrogen, ratio of carbon to nitrogen, pH, minerals, level of spawning, and surfactant. Extrinsic factors are also relevant, such as temperature, humidity, luminosity and air composition, and envase [14]. Whether grown in the field or in a controlled environment, the growing process for each mushroom species has its own characteristics and environmental implications. Some findings have shown that mushrooms cultivated in a controlled environment outperforms growing in the field, as these conditions often enhance their nutritional, chemical, and bioactive composition [6,15,16].

The C/N ratio is a particularly important factor in mushroom production, and to obtain profitable mushroom yields, the C/N ratio of the substrate must be controlled. Most agricultural residues are defined as materials with a low nitrogen content, and in this sense, the supplementation of organic nitrogen or inorganic nitrogen into substrates is essential. Mushrooms can grow on numerous types of substrates, but the rates of substrate utilization and mushroom growth are dependent on the mushroom species [17]. Raw materials used for the production of the *A. subrufescens* compost are rich in Gram-negative and Gram-positive bacteria, the most common being *Pseudomonas* and *Bacillus*. This community varies throughout the composting process and at the end of the substrate composting process, the presence of *B. licheniformis* and *B. megaterium* were verified [18]. This bacterial community play important roles in the assimilation and transformation of ammonia compost [19], exerting a positive effect on the fructification of mushrooms [20].

On the other hand, the strains used in experimental crops in recent years came from Brazil, France, Spain, the USA, Mexico, Taiwan, Belgium, and Italy. Studies have been conducted mainly with strains isolated from commercial crops (Brazil), with several strains collected from the wild (France, Spain, Thailand, and others) [13].

Therefore, mushroom cultivation requires both scientific knowledge and practical experience. In this sense, understanding cultivations conditions could significantly benefit mushroom science, and support the development of strategies that would enable scientists to increase both productivity and mushroom quality [17].

In this sense, the purpose of the present work was to evaluate the influence of cultivation process (in the field and under controlled conditions) in the nutritional value (proximal composition), chemical parameters (free sugars, organic acids, fatty acids, tocopherols, phenolic acids and related compounds), and the bioactive properties (antioxidant and antimicrobial activities) by using commercial strains of *A. subrufescens*.

## 2. Materials and Methods

### 2.1. Mushroom Samples and Cultivation Conditions

Six commercial Brazilian strains of *A. subrufescens* were used: AS CS7 (acquired from the Federal University of Lavras, Brazil—MW200295.2 GenBank number); AS 18/01 (isolated from a grower in the region of São Paulo, Brazil—MW200293.2 GenBank number); AS 98/11 (isolated from growers in the region of Mogi das Cruzes, Brazil—MW200294.2 GenBank number); AS 16/01 (isolated from commercial spawn lab in Valinhos, Brazil—MW200292.1 GenBank number); AS 04/49 (isolated from a grower in the region of São José do Rio Preto, Brazil—MW89464.7 GenBank number); AS 19/01 (isolated from a series of crops carried out at CECOG/UNESP, in the city of Dracena, Brazil—MWMW89464.8 GenBank number).

Briefly, mixtures of various agricultural materials were used in mushroom controlled cultivation such as bulky plant-derived materials (*Panicum maximum*, sugarcane bagasse and soybean) and chemicals (urea, ammonium sulfate, simple superphosphate and limestone).

The abbreviation AS stands for *A. subrusfescens*, followed by the year of collection of the isolate and the collection number, either 98 (year of collection) or 11 (number of collection).

**Figure 1:**
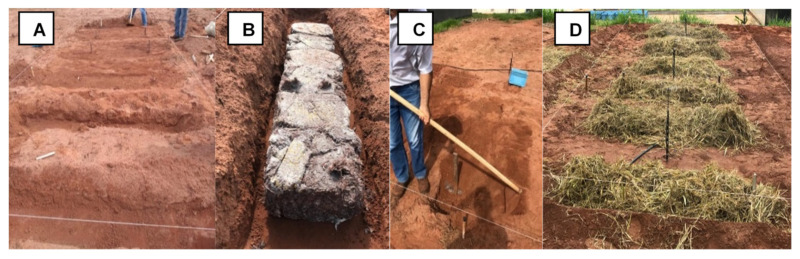
*Agaricus subrufescens* produced in the field: (**A**) furrow opening in the soil; (**B**) addition of the colonized compost in the furrow; (**C**) covering the compost with the same and soil removed; (**D**) covering the casing layer with straw (Junior et al. [16]).

**Figure 2:**
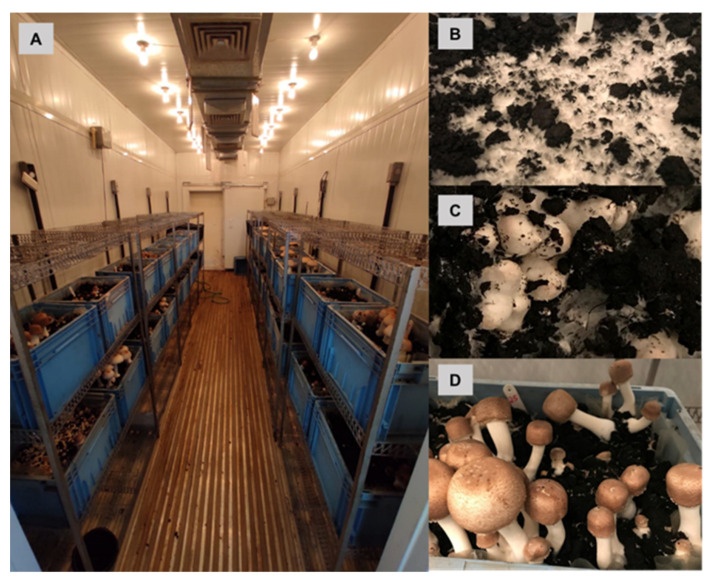
*Agaricus subrufescens* produced in a controlled environment: (**A**) specific climatic chamber for mushroom production; (**B**) mycelium development in peat moss; (**C**) formation of beginnings; (**D**) mushrooms in the harvest phase.

**Figure 3:**
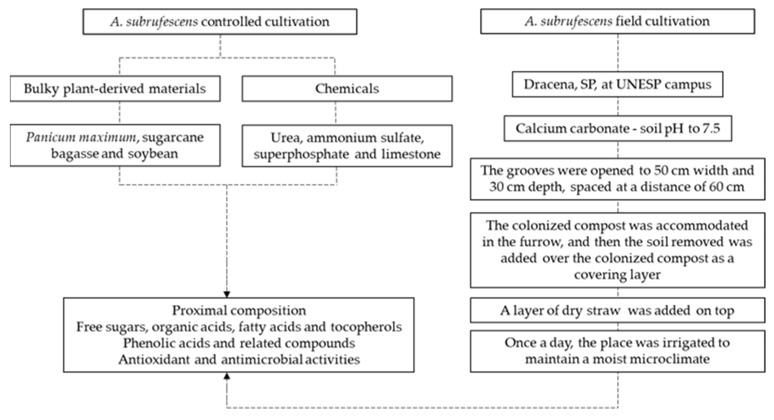
An illustration of the conditions used for *Agaricus subrufescens* production.

The strains, spawn production, compost, inoculation and mycelium run, cultivation in the field (Figure 1), and cultivation in the controlled chamber (Figure 2) conditions were described in detail by Junior et al. [16]. The scheme conditions used for the cultivation of mushrooms are shown in Figure 3.

### 2.2. Proximal Composition

The samples were analyzed for proximal composition (protein, fat, carbohydrates, and ash) according to the Official Methods of Analysis of AOAC [21]. Crude protein was estimated by the macro-Kjeldahl method (N × 4.38), via an automatic distillation and titration unit (model Pro-Nitro-A, JP Selecta, Barcelona, Spain). Soxhlet extraction was employed to access the crude fat, with petroleum ether throughout 7 h. The ash content was determined by incineration at 550 ± 10 °C. Total carbohydrates were calculated by difference: Total carbohydrates (g/100 g of dried weight (dw)) = 100 − (fat + g ash + g proteins). Total energetic value was calculated according to the Atwater system using the formula: Energy (kcal/100 g dw) = 4 × (g protein + g carbohydrates) + 9 × (g fat).

### 2.3. Chemical Composition Regarding Hydrophilic Compounds

#### 2.3.1. Free Sugars

Free sugars extraction from the dry samples was performed according to Spréa et al. [22]. The compounds were identified by high performance liquid chromatography with a refraction index detector (HPLC-RI; Knauer, Berlin, Germany, Smartline 1000 and Smartline 2300 systems, respectively), as previously described by the authors. Peaks identification was carried out by comparisons of their relative retention time (Rt) with authentic standard. Quantification was finished using raffinose as internal standard (IS; Sigma Aldrich, St. Louis, MO, USA), and with calibration curves constructed from authentic standards. Results were managed in a Clarity Software (Data Apex, Prague, Czech Republic) and expressed in g per 100 g of dw.

#### 2.3.2. Organic Acids

Organic acids were determined following a procedure previously described and optimized by the authors [23]. Briefly, samples (~1.5 g) were extracted by stirring with 25 mL of metaphosphoric acid (25 °C at 150 rpm) for 25 min and subsequently filtered through Whatman No. 4 paper. The assessment was achieved by ultra-fast liquid chromatography coupled to a photodiode array detector (UFLC-PDA; Shimadzu Corporation, Kyoto, Japan). The identification was carried out by comparing the chromatograms obtained for the analyzed samples with those obtained using commercial standards. The quantification of the compounds was completed by relating the peak areas, recorded at 215 nm, with the calibration curves obtained with commercial standards for each compound. The results were expressed in g per 100 g of dw.

### 2.4. Chemical Composition Regarding Lipophilic Compounds

#### 2.4.1. Fatty Acids

Fatty acid methyl esters (FAME) were investigated after trans-esterification of the lipid fraction obtained through Soxhlet extraction as previously described by the authors [24], and determined by gas-liquid chromatography with flame ionization detection, using a YOUNG IN Crhomass 6500 GC System instrument equipped with a *split/splitless* injector, a flame ionization detector (FID) and a Zebron-Fame column. Fatty acids identification and quantification was performed by comparing the relative retention times of FAME peaks from samples with standards and results were recorded and processed using the Software Clarity DataApex 4.0 Software (Prague, Czech Republic) and expressed in relative percentage of each fatty acid.

#### 2.4.2. Tocopherols

Tocopherols were determined following a procedure previously described by the authors [22]. The formerly described HPLC system, coupled to a fluorescence detector (FP-2020; Jasco, Tokyo, Japan) programmed for excitation at 290 nm and emission at 330 nm, was used. The compounds were identified by chromatographic comparisons with authentic commercial standards, and quantification was based on the response of the fluorescence signal, using the internal standard method and by chromatographic comparison with standards. Tocol was used as internal standard, and the results were expressed in µg per 100 g of dw.

### 2.5. Phenolic Acids and Related Compounds

Each sample (~1.5 g) was extracted with methanol:water (80:20, *v/v*; 30 mL) at −20 °C for 6 h. After sonication for 15 min and filtered through Whatman No. 4 paper, the residue was then extracted with two additional 30 mL portions of the methanol:water mixture, and combined extracts were evaporated at 40 °C under reduced pressure to remove methanol. The aqueous phase was submitted to a liquid–liquid extraction with diethyl ether (3 × 30 mL) and ethyl acetate (3 × 30 mL). Anhydrous sodium sulphate was added to the combined organic phases and after were filtrated through Whatman No. 4 paper, evaporated to dryness and re-dissolved in a mixture of water:methanol (80:20, *v/v*; 1 mL). The obtained extracts were filtered through a 0.22 μm disposable LC filter disk for HPLC analysis. Phenolic acids and related compounds were determined in the UFLC system mentioned above, as previously described by the author [25]. DAD detection was carried out using 280 as preferred wavelengths. The phenolic acids and related compounds were quantified by comparison of the area of their peaks with calibration curves obtained from commercial standards of each compound. The results were expressed in μg per g dw.

### 2.6. Bioactive Properties

#### 2.6.1. Antioxidant Activity Evaluation

For the thiobarbituric acid reactive substances (TBARS) assays, the mushrooms hydromethanolic extract was re-dissolved in methanol/water (80/20, *v/v*), and subjected to dilutions from 20 mg/mL to 0.625 mg/mL. Porcine brain cells (Sus scrofa), were used as subtract, according to previously described procedure [26]. The results were expressed in EC_50_ values (mg/mL), which represents the extract concentration (mg/mL) necessary to prevent 50% of lipid peroxidation in porcine brain cells. The antihemolytic potential of the hydromethanolic extracts re-dissolved in PBS was assessed through the oxidative hemolysis inhibition assay (OxHLIA), as mentioned in detailed [27]. The results were expressed as IC_50_ values for Δ*t* of 60 and 120 min (extract concentration required to keep 50% of the erythrocyte population intact in the time mentioned, µg/mL). Trolox (Sigma Chemical Co., St. Louis, MO, USA) was used as positive control in both assays.

#### 2.6.2. Antimicrobial Activity

The antibacterial activity was evaluated by the broth microdilution method coupled to the rapid *p*-iodonitrotetrazolium chloride (INT) colorimetric assay [28]. The tested microorganisms were clinical isolates from patients hospitalized in various departments of the Local Health Unit of Bragança and Hospital Center of Trás-os-Montes and Alto-Douro Vila Real, Northeast of Portugal, and included three Gram-positive (*Enterococcus faecalis*, *Listeria monocytogenes*, and methicillin resistant *Staphylococcus aureus*) and five Gram-negative (*Escherichia coli*, *Klebsiella pneumoniae*, *Morganella morganii*, *Proteus mirabilis*, and *Pseudomonas aeruginosa*) bacteria. The minimum inhibitory concentration (MIC) was determined by the colorimetric microbial viability based on the reduction of the INT colorant (0.2 mg/mL; Panreac AppliChem, Barcelona, Spain). The samples were first of all dissolved in 5% (*v/v*) Dimethyl sulfoxide (DMSO) and 95% of autoclaved distilled water to give a final concentration of 20 mg/ mL for the stock solution. Then, 90 μL of this concentration was added in the first well (96-well microplate) in duplicate with 100 μL of Tryptic Soy Broth (TSB). In the remaining wells 90 μL of TSB medium were added. Then, the samples were serially diluted to obtain the concentration ranges (20 to 0.3 mg/mL). To finish, 10 μL of inoculum (standardized at 1.5 × 10^6^ Colony Forming Unit (CFU) /mL) was added at all the well assuring the presence of 1.5 × 10^5^ CFU. Two negative controls were prepared, one with TSB and another one with the extract. Antibiotics were used as negative controls, namely ampicillin (20 mg/mL) and imipenem (1 mg/mL) for Gram-negative bacteria, and vancomycin (1 mg/mL) and ampicillin (20 mg/mL) for Gram-positive bacteria. The microplates were incubated at 37 °C for 24 h. The MIC of samples was detected following addition (40 μL) of 0.2 mg/mL *p*-iodonitrotetrazolium chloride (INT) and incubation at 37 °C for 30 min. MIC was defined as the lowest concentration that inhibits the visible bacterial growth determinate by changing the coloration from yellow to pink if the microorganisms are viable. For the determination of MBC, 10 μL of liquid from each well that showed no change in color was plated on solid medium, blood agar (7% sheep blood), and incubated at 37 °C for 24 h. The lowest concentration that yielded no growth determined the MBC. MBC was defined as the lowest concentration required to kill bacteria.

### 2.7. Statistical Analysis

The experiments were carried out with six treatments (strains) of *A. subrufescens* under two environmental culture conditions. All analyses were conducted in triplicate, and results were presented as mean ± standard deviation (SD) (except for antibacterial activity). The results were analyzed by a one-way analysis of variance (ANOVA) followed by a Tukey’s HSD test, with α = 0.05. The fulfilment of the ANOVA requirements was tested using Shapiro–Wilk to assess the normality.

## 3. Results and Discussion

### 3.1. Proximal Composition

The strain, temperature, humidity, exposure to sunlight, and the interaction with other microorganisms are some factors that can influence the productivity of *A. subrufescens*, and consequently, the production of the important metabolites in this species and responsible for its biological properties.

The results in Table 1 present the nutritional composition of *A. subrufescens*, determined by the content of fat, protein, ash, carbohydrates, and the energetic value in two different environmental conditions of cultivation (field and controlled environment). Both in the field and in a controlled environment, carbohydrates were the main macronutrients found in all samples (59 to 72.6 g/100 g dw), followed by proteins (18.8 to 35.8 g/100 g dw), ash (8.1 to 10.66 g/100 g dw), and fat, which presented the lowest values (1.42 to 1.96 g/100 g dw). In general, the amount of carbohydrates present in each strain was slightly higher in controlled environment than in the field (except for the AS 16/01, 53.4 g/100 g dw), with the strain AS 19/01 presenting the highest content in both cultivation conditions (field and controlled environment, 68.11 and 72.6 g/100 g dw, respectively).

Regarding proteins content, in general, the strains grown in the field had a slightly better performance than the ones cultivated in a controlled environment, except for AS 16/01, which stood out in the cultivation in a controlled environment (35.8 g/100 g dw). AS 16/01 was also the strain that presented the highest amount of ash in the two conditions (field and controlled environment, 10.66 and 9.0 g/100 g dw, respectively). In the analysis of the fat content, it was possible to notice that the strains cultivated in a controlled environment presented relatively lower contents of fat than those cultivated in the field, except for the strain AS 19/01, which presented the highest values in the controlled-environment cultivation (1.6 g/100 g dw). Lastly, the energetic contribution obtained for the strains cultivated in controlled environment were slightly higher than those obtained for the strains cultivated in the field, a result most influenced by the carbohydrates content of each strain (373 to 380 g/100 g dw).

The high content of proteins and carbohydrates and the low content of fat present in the mushrooms indicate that they can be a good option to be included as a food source in human diet. Additionally, the amount of ash represents a good concentration of micronutrients, which are essentials for the proper functioning of the human body [8]. These results are in a similar range with a study performed by Carneiro et al. [29], who investigated the chemical composition of dried powder formulations of *A. blazei* used as a food supplement capsule in Brazil. In this study, the authors found similar amounts for the same evaluated parameters, namely, carbohydrates (59.42 g/100 g dw) the major macronutrients found in samples analyzed, followed by protein (31.29 g/100 g dw), ash (7.47 g/100 g dw), fat (1.82 g/100 g dw) and energy (379.24 kcal/100 g dw), as well as in our study. Similar results have been also described for mushrooms from *Agaricus* genus distributed and cultivated in India [30].

### 3.2. Chemical Composition Regarding Hydrophilic Compounds

Free sugars were also analyzed and the results are present in Table 1. Glucose and trehalose were the sugars found in higher amounts in all the evaluated samples, followed by fructose, present in smaller quantities. The fructose content in strains grown in the field was shown to be higher than in strains grown in a controlled environment, except for the strain AS 16/01, which presented the highest values in the controlled-environment cultivation (0.270 g/100 g dw). The opposite occurred with glucose and trehalose, which appeared in higher amounts in strains grown in a controlled environment than in those grown in the field, thereby influencing the total sugars value. AS 19/01 strain presented the highest content of glucose (field and controlled environment, 29.6 and 39.2 g/100 g dw, respectively) and trehalose (field and controlled environment, 2.06 and 3.7 g/100 g dw, respectively), as well as the highest values of total sugars for both cultivation environments (field and controlled environment, 31.9 and 43.1 g/100 g dw, respectively). Cho et al. [31] also identified trehalose in strains of *A. brasiliensis* cultivated by the Korean conventional method (2.60 g/100 g dw), along with mannitol (21.80 g/100 g dw). In their study, Tsai et al. [32] analyzed the sugar profile of artificially cultivated *A. blazei* from Taiwan, detecting mannitol (7.94 g/100 g dw) as the main sugar identified, followed by trehalose (2.98 g/100 g dw) and glucose (2.76 g/100 g dw), which was the one present in smaller quantities.

With respect to the assessment of organic acids, all the strains presented oxalic, malic, citric, and fumaric acids, and the results are presented in Table 1. Malic and citric acids were the ones found in higher concentrations, followed by oxalic and fumaric, with fumaric being found just in traces. Strains grown in the field presented slightly higher values of total organic acids when compared to strains grown in a controlled environment, with this parameter being most influenced by the quantity of malic and citric acid, in both conditions. The AS 18/01 strain presented the highest amount of total organic acids among those cultivated in the field (48.0 g/100 g dw), while AS CS7 was the one that stood out among those cultivated in a controlled environment (42.0 g/100 g dw). In the research conducted by Carvajal et al. [33], among the organic acids identified in fruiting bodies and submerged culture mycelia of *A. brasiliensis*, citric acid was detected as the main one (117.9 g/100 g dw), followed by malic (21.7 g/100 g dw), oxalic (11.5 g/100 g dw), and in lower concentrations, fumaric acid (0.8 g/100 g dw), which are similar results to the ones obtained in the present work. Gąsecka et al. [34] also evaluated the organic acids profile of *A. subrufescens* from Poland, cultivated in a controlled environment, describing the presence of oxalic (54.7 g/100 g dw) and malic acid (1.61 g/100 g dw), as in our study; lactic acid (133.6 g/100 g dw) was also identified, being the one found in higher amounts. The organic acids profile is responsible for organoleptic properties of the mushroom, such as taste and flavor, and can also play a biological role, due to their antioxidant, acidifying, neuroprotective, anti-inflammatory, and antimicrobial properties [35].

### 3.3. Chemical Composition Regarding Lipophilic Compounds

The evaluation of the fatty acid profile identified 16 different compounds (Table S1), of which, the most abundant were linoleic (C18:2n6c), palmitic (C16:0), stearic (C18:0) and behenic (C22:0) acids. Considering the classification of fatty acids, polyunsaturated fatty acids (PUFA) were found in the highest concentrations in both cultivation environments, followed by saturated fatty acids (SFA) and, in lower concentrations, monounsaturated fatty acids (MUFA) (Table 2). The strain AS 98/11 stood out between the others in terms of PUFA content, showing the highest amounts in the field (73.6%) and in a controlled environment (73.0%). The prevalence of PUFA, mainly due to the contribution of linoleic acid (75.9%), was also observed in the research conducted by Cho et al. [31], which analyzed the chemical composition of fruiting bodies of cultivated *A. brasiliensis*. Similar results were found by Carneiro et al. [29], on dried powder formulations of *Agaricus blazei* (73.58%). Previous studies indicate that PUFA have an important role in several functions of human body, regulating inflammation, immunity, blood vessels, cellular growth, pain, and sleep. They are also especially important to regulate cell membrane fluidity and for the development, maintenance, and function of the nervous system [35].

**Figure 4:**
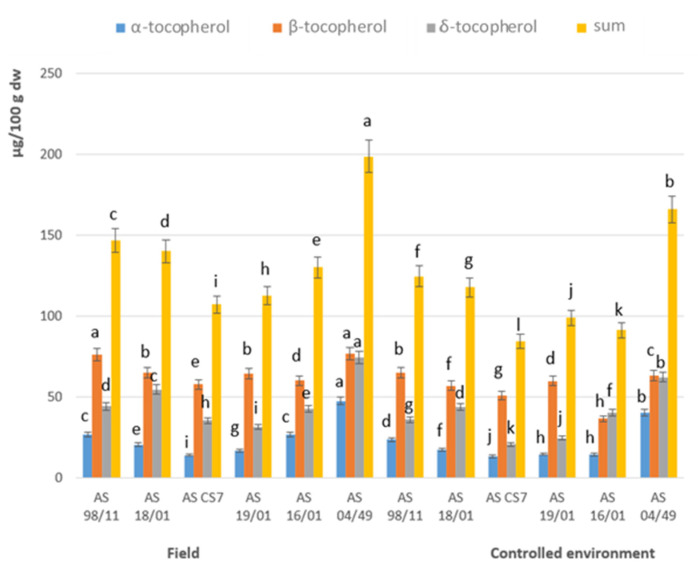
Content of tocopherols of the studied Agaricus subrufescens samples produced in the field and in controlled en-vironment crops. Mean values (*n* = 3); identical superscripts (a–l) denote no significant (*p* < 0.05) difference between mean values in columns according to Tukey’s HSD test (ANOVA) for mushrooms produced in the field and in controlled environment crops.

With respect to the assessment of tocopherols (Figure 4), the isoforms α, β, and δ-tocopherol were distinguished in all strains analyzed and in both cultivation conditions, where β- and δ-tocopherol were the main isoforms identified. In general, the amount of each isoform was slightly higher in field cultivation when compared to cultivation in a controlled environment, with the AS 04/49 strain having the highest sum of tocopherols in both cultivation conditions (field and controlled environment, 198.6 and 11.6 g/100 g dw, respectively) (Table S2). Tsai et al. [36] evaluated the biochemical composition of air-dried *A. blazei* mushroom commercialized in Taiwan, and identified α-tocopherol and δ-tocopherol as in our study, along with γ-tocopherol. Carneiro et al. [29] also studied the tocopherol profile of dried powder formulations of *A. blazei*, showing the presence, in higher amounts of α-tocopherol (77.79 µg/100 g dw) and followed also by γ-tocopherol (46.47 µg/100 g dw). The sum of total tocopherols (124.25 µg/100 g dw) found for *A. blazei* in the referred study was similar than the values found in the present work (field and controlled environment, 107.0 to 198.6 µg/100 g dw and 84.4 to 166 µg/100 g dw, respectively, Table S2). Tocopherols are important antioxidant agent due to its capacity to inhibit lipid autoxidation by scavenging free radicals and by reacting with singlet oxygen. The α- isoform is generally considered as the most effective antioxidant, followed by β- and γ-tocopherol, and then δ-tocopherol [37,38].

### 3.4. Phenolic Acids and Related Compounds

**Figure 5:**
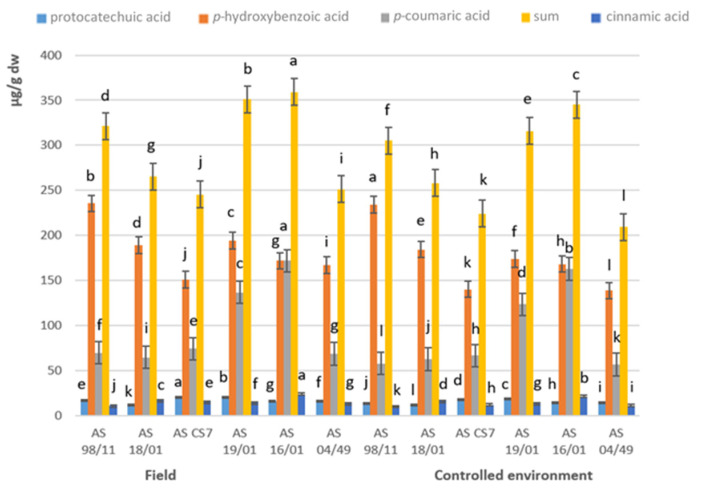
Content of phenolic acids and related compounds of the studied *Agaricus subrufescens* samples produced in the field and in controlled environment crops. Mean values (*n* = 3); identical superscripts (a–l) denote no significant (*p* < 0.05) difference between mean values in columns according to Tukey’s HSD test (ANOVA) for mushrooms produced in the field and in controlled environment crops.

Regarding phenolic acids and related compounds (Figure 5 and Table S2), the results obtained by the chromatographic analysis revealed the presence of *p*-hydroxybenzoic acid, this one in higher quantities in all strains, followed by protocatechuic, cinnamic, and *p*-coumaric acids. The strain AS 98/11 presented the highest amounts of *p*-hydroxybenzoic acid, both in the field and in a controlled environment (235.5 and 234 µg/g dw, respectively), while strain AS 16/01 was the one that presented higher sum of the total phenolic compounds in the two cultivation conditions (field and controlled environment, 359.0 and 345 µg/g dw, respectively), most influenced by *p*-hydroxybenzoic and *p*-coumaric acids (Table S2). Carneiro et al. [29] detected the presence of *p*-hydroxybenzoic (0.64 µg/g dw), trans-*p*-coumaric (0.08 µg/g dw), and cinnamic acids (0.05 µg/g dw) in dried powder formulations of *A. blazei* from Brazil. Gąsecka et al. [34] detect the presence of gallic, caffeic, *p*-hydroxybenzoic, *p*-coumaric, ferulic, chlorogenic, syringic, trans-cinnamic and protocatechuic acids in *A. brasiliensis* cultivated in controlled environment in Poland, being the gallic and trans-cinnamic the main ones.

### 3.5. Bioactive Properties

The antioxidant activity of the samples was determined by the evaluation of its capacity to avoid lipid peroxidation of porcine brain tissues (TBARS) and hemolysis of sheep blood cells (OxHLIA), and the results obtained are shown in Table 3. Regarding TBARS assay, both strains cultivated in the field and controlled environment present relevant antioxidant activity, giving EC_50_ values between 0.44 to 1.91 mg/mL for the strains cultivated in the field, and 0.80 to 1.90 mg/mL for those grown in a controlled environment. Stojković et al. [39] also analyzed the antioxidant activity of ethanolic extracts obtained from *A. subrufescens* commercial strains though TBARS method, and the results revealed an average EC_50_ of 1.80 mg/mL, consistent with the result obtained in the present research work.

The results of the antioxidant activity evaluated by the OxHLIA method are also presented in Table 3. The concentrations required to protect half of the erythrocyte population from the hemolytic action triggered by oxidative agent (IC_50_) for 60 min ranges from 88 µg/mL to 221 µg/mL for the strains grown in the field, and 83 µg/mL to 310 µg/mL for cultivation in controlled environment.

In general, the strains AS 18/01 and AS CS7 cultivated in the field showed better performance in both essays, while AS 16/01 and AS 04/49 did not present significant variation between the cultivation environments.

There are other well-known methods used to evaluate the antioxidant potential of an extracts, but most of them are chemical assays founded on scavenging of free radicals [40]. As an example, DPPH assay is a method that measures the scavenging ability of antioxidant substances towards DPPH radical. Wei et al. [40] assessed the antioxidant activity of *A. blazei* ethanolic extract using the DPPH assay, and the results obtained revealed relevant antioxidant activity (IC_50_; 0.5 mg/mL).

The antioxidant activity exhibited by the strain extracts can be related to the presence of tocopherols, organic acids, and phenolic compounds found in each sample. Several research works describe the antioxidant potential of these substances in different mushrooms species [29,34,41,42]. The results obtained show that the mushrooms content of tocopherols, organic acids, and phenolic compounds make them valuable food sources, having a great potential to be used to prevent oxidative damage.

Table 4 shows the results of the antimicrobial activity obtained by hydromethanolic extract of *A. subrufescens* against three Gram-positive and five Gram-negative pathogenic strains, isolated from hospitalized patients. In general, all extracts present moderated activity against the bacterial strains tested in this study, with strain AS 19/01 being the one that presented lower MIC values against E. coli and all the Gram-positive bacteria in both environmental conditions and, therefore, better antimicrobial activity when compared with the other strains. Mazzutti et al. [43] evaluated the antimicrobial activity of supercritical fluid extraction from cultivated *A. brasiliensis* against Gram-positive bacteria (*S. aureus* and *B. cereus*) and Gram-negative bacteria (*E. coli* and *P. aeruginosa*), and the results obtained showed that the extracts were efficient as inhibitors of the microorganism growth for Gram-positive bacteria, but not against Gram-negative, associating this fact to the cell wall properties. The external membrane of Gram-negative bacteria renders highly hydrophilic surfaces, whereas the negative charge of the surface of the Gram-positive wall may reduce their resistance to antibacterial compounds.

Stojković et al. [39] also described efficient antimicrobial activity of ethanolic extract from *A. brasiliensis* against Gram-positive bacteria (*S. aureus*, *B. cereus*, *Micrococcus flavus* and *L. monocytogenes*), and Gram-negative bacteria (*P. aeruginosa*, *Salmonella typhimurium*, *E. coli*, and *Enterobacter cloacae*). The antimicrobial properties of mushrooms are also closely linked to its composition, being most influenced by the presence of phenolic compounds [44].

## 4. Conclusions

The present work provides new insights concerning the biosynthesis of biochemical parameters by different strains of mushrooms growing under two environmental cultivation conditions.

The results obtained in this study demonstrate that *A. subrufescens* can be well-adapted to the cultivation in a controlled environment, depending on the strains and the conditions, and the variables found between the cultivation systems applied in this work may help to elucidate the conditions necessary for the modulation of the production of its metabolites content.

The differences between strains and the two cultivation conditions can indicate that the production in a controlled environment may interfere in the composition of the mushrooms, since strains cultivated in the field presented higher levels for most of the parameters evaluated in this work. The composition of cellulose, hemicellulose, and lignin in lignocellulose materials depends on the plant species, and this is an important factor that can impact mushroom cultivation.

Furthermore, in recent years, research studies have tackled the adaptability of *A. subrufescens* to commercial cultivation in the main areas of production of edible mushrooms. This work presents an integrated approach, helps predict behavior and encourages the field cultivation of *A. subrufescens,* a promising source of biochemical compounds for the food and pharmaceutical industries.

## Figures and Tables

**Table 1:** Nutritional, energetic value and hydrophilic compounds of the studied *Agaricus subrufescens* samples produced in the field and in controlled environment crops.

	Field						Controlled Environment					
	AS 98/11	AS 18/01	AS CS7	AS 19/01	AS 16/01	AS 04/49	AS 98/11	AS 18/01	AS CS7	AS 19/01	AS 16/01	AS 04/49
Nutritional value (g/100 g dw)												
Fat	1.96 ± 0.03 a	1.8 ± 0.1 b	1.54 ± 0.05 d	1.42 ± 0.04 e	1.9 ± 0.1 a	1.94 ± 0.02 a	1.47 ± 0.02 e	1.6 ± 0.1 c	1.44 ± 0.04 e	1.6 ± 0.1 c	1.73 ± 0.01 b	1.93 ± 0.04 a
Proteins	29 ± 1 b	26.8 ± 0.1 c	27 ± 1 c	22.3 ± 0.2 g	28.83 ± 0.04 b	25.0 ± 0.1 e	26.7 ± 0.4 c	22.46 ± 0.01 g	24.5 ± 0.4 f	18.8 ± 0.2 h	35.8 ± 0.1 a	25.6 ± 0.2 d
Ash	10 ± 1 b	9.4 ± 0.1 c	9.3 ± 0.3 c	8.1 ± 0.2 f	10.66 ± 0.03 a	8.67 ± 0.05 e	8.4 ± 0.3 f	7.2 ± 0.3 h	7.5 ± 0.2 g	7.13 ± 0.02 h	9.0 ± 0.3 d	7.3 ± 0.2 gh
Carbohydrates	59 ± 1 i	62.0 ± 0.1 h	62.3 ± 0.6 h	68.11 ± 0.03 c	58.6 ± 0.1 j	64.4 ± 0.1 f	63.4 ± 0.1 g	68.8 ± 0.1 b	66.6 ± 0.5 d	72.6 ± 0.2 a	53.4 ± 0.3 k	65.2 ± 0.1 e
Energy (Kcal/100 g dw)	368 ± 2 g	371 ± 1 f	370 ± 1 f	375 ± 1 c,d	367.0 ± 0.5 h	375.0 ± 0.1 c	374 ± 1 d	380 ± 1 a	377.1 ± 0.5 b	379.3 ± 0.3 a	373 ± 1 e	380 ± 1 a
Free sugars (g/100 g dw)												
Fructose	0.270 ± 0.004 b	0.280 ± 0.008 a	0.280 ± 0.02 a	0.220 ± 0.004 e	0.240 ± 0.004 d	0.260 ± 0.004 c	0.170 ± 0.02 g	0.210 ± 0.003 f	0.240 ± 0.003 d	0.170 ± 0.006 g	0.270 ± 0.005 b	0.220 ± 0.002 e
Glucose	19.6 ± 0.2 i	25.8 ± 0.1 h	28.3 ± 0.2 f	29.6 ± 0.3 e	18.6 ± 0.1 j	16.8 ± 0.5 k	27.8 ± 0.4 g	31.66 ± 0.02 c	34.1 ± 0.3 b	39.2 ± 0.3 a	27.6 ± 0.1 g	30.8 ± 0.5 d
Trehalose	1.03 ± 0.01 e	0.950 ± 0.002 f	0.90 ± 0.01 g	2.06 ± 0.02 b	0.79 ± 0.02 i	0.21 ± 0.01 j	0.8 ± 0.1 h,i	1.21 ± 0.04 d	0.87 ± 0.02 g, h	3.7 ± 0.1 a	1.5 ± 0.1 c	0.85 ± 0.02 g,h
Sum	20.9 ± 0.2 h	27.1 ± 0.1 g	29.5 ± 0.2 e	31.9 ± 0.2 d	19.63 ± 0.04 i	17.3 ± 0.5 j	28.8 ± 0.5 f	33.09 ± 0.02 c	35.2 ± 0.3 b	43.1 ± 0.4 a	29.4 ± 0.2 e	31.9 ± 0.5 d
Organic acids (g/100 g dw)												
Oxalic acid	2.71 ± 0.07 e	2.75 ± 0.01 e	2.509 ± 0.003 f	1.83 ± 0.04 i	4.4 ± 0.1 a	3.07 ± 0.04 c	2.2 ± 0.1 h	2.53 ± 0.03 f	2.32 ± 0.03 g	1.77 ± 0.02 j	3.96 ± 0.01 b	2.80 ± 0.02 d
Malic acid	24.0 ± 0.2 c	26.2 ± 0.2 b	27.52 ± 0.02 a	18.2 ± 0.3 f	3.6 ± 0.1 h	3.22 ± 0.03 k	19.7 ± 0.1 d	19.3 ± 0.1 e	23.9 ± 0.2 c	14.4 ± 0.1 g	3.42 ± 0.03 i	3.05 ± 0.03 j
Citric acid	16.42 ± 0.01 c	19.0 ± 0.1 a	16.6 ± 0.1 b	12.33 ± 0.05 k	16.0 ± 0.2 d	14.2 ± 0.2 h	14.6 ± 0.2 f	14.38 ± 0.01 g	15.75 ± 0.03 e	10.7 ± 0.1 l	13.8 ± 0.1 j	13.98 ± 0.04 i
Fumaric acid	tr	tr	tr	tr	tr	tr	tr	tr	tr	tr	tr	tr
Sum	43.2 ± 0.2 c	48.0 ± 0.4 a	46.7 ± 0.1 b	32.3 ± 0.2 g	24.0 ± 0.3 i	20.5 ± 0.1 k	36.5 ± 0.4 e	36.2 ± 0.1 f	42.0 ± 0.3 d	26.9 ± 0.2 h	21.2 ± 0.1 j	19.83 ± 0.02 l

Results are expressed mean ± SD (*n* = 3); dw—dry weight, tr—traces. In each line, different letters indicate significant differences (*p* < 0.05) between samples. Free sugars calibration curves: fructose (y = 1.04x; *R*^2^ = 0.999; LOD = 0.05 mg/mL; LOQ = 0.18 mg/mL), glucose (y = 0.935 x; *R*^2^ = 0.999; LOD = 0.08 mg/mL; LOQ = 0.25 mg/mL), and trehalose (y = 0.991x; *R*^2^ = 0.999; LOD = 0.07 mg/mL; LOQ = 0.24 mg/mL). Organic acids calibration curves: oxalic acid (y = 9E + 106x + 459.731; *R*^2^ = 0.994; LOD = 12.55 μg/mL; LOQ = 41.82 μg/mL); malic acid (y = 912.441x + 92.665; *R*^2^ = 0.999; LOD = 35.76 μg/mL; LOQ = 119.18 μg/mL); citric acid (y = 1E + 106x + 45.682; *R*^2^ = 1; LOD = 10.47 μg/mL; LOQ = 34.91 μg/mL) and fumaric acid (y = 1E + 0.8x + 614.399; *R*^2^ = 1; LOD = 0.08 μg/mL; LOQ = 0.26 μg/mL).

**Table 2:** Main fatty acids composition of the studied *Agaricus subrufescens* samples produced in the field and in controlled environment crops.

	Field							Controlled Environment				
	AS 98/11	AS 18/01	AS CS7	AS 19/01	AS 16/01	AS 04/49	AS 98/11	AS 18/01	AS CS7	AS 19/01	AS 16/01	AS 04/49
Fatty Acids (%)												
C16:0	12.7 ± 0.2	15 ± 1	14.3 ± 0.1	17.60 ± 0.03	13.5 ± 0.1	13.4 ± 0.3	12.31 ± 0.01	15.0 ± 0.1	17 ± 1	16.5 ± 0.2	15 ± 1	14 ± 1
C18:0	3.8 ± 0.1	4.9 ± 0.3	4.42 ± 0.07	4.97 ± 0.02	5.0 ± 0.1	4.4 ± 0.2	4.250 ± 0.001	4.6 ± 0.3	5.23 ± 0.01	5.15 ± 0.05	4.9 ± 0.3	4.9 ± 0.3
C18:1n9c	1.2 ± 0.1	2.1 ± 0.2	1.54 ± 0.03	1.5 ± 0.1	1.6 ± 0.1	1.28 ± 0.03	1.19 ± 0.03	1.5 ± 0.1	1.9 ± 0.1	2.0 ± 0.1	1.6 ± 0.1	1.4 ± 0.1
C18:2n6c	73.4 ± 0.2	68 ± 1	66.2 ± 0.2	64 ± 1	70.0 ± 0.3	71 ± 1	72.8 ± 0.1	68.1 ± 0.1	64 ± 1	64 ± 1	67 ± 2	67.5 ± 0.4
C20:0	1.48 ± 0.01	1.69 ± 0.03	1.38 ± 0.01	1.69 ± 0.01	1.7 ± 0.1	1.7 ± 0.1	1.50 ± 0.01	1.34 ± 0.04	1.83 ± 0.03	1.7 ± 0.2	1.6 ± 0.1	1.6 ± 0.1
C22:0	2.83 ± 0.03	3.1 ± 0.1	3.06 ± 0.04	3.1 ± 0.1	3.08 ± 0.05	3.04 ± 0.02	3.19 ± 0.03	3.3 ± 0.1	3.3 ± 0.1	3.5 ± 0.3	3.06 ± 0.03	3.5 ± 0.3
SFA	24.7 ± 0.3 i	29 ± 1 d	31.2 ± 0.2 b	33 ± 1 a	27.8 ± 0.2 f	27 ± 1 g	25.3 ± 0.1 h	29.7 ± 0.2 c	33 ± 1 a	33 ± 1 a	30 ± 2 b	30.4 ± 0.4 b,c
MUFA	1.7 ± 0.1 i	2.8 ± 0.2 a	2.44 ± 0.01 c	2.2 ± 0.1 d	2.1 ± 0.1 e	1.8 ± 0.1 h	1.62 ± 0.04 j	2.0 ± 0.2 f	2.2 ± 0.1 d	2.6 ± 0.1 b	2.1 ± 0.1 e	1.94 ± 0.03 g
PUFA	73.6 ± 0.2 a	68 ± 1 c	66.3 ± 0.2 e	65 ± 1 f	70.1 ± 0.3b	71 ± 1 b	73.0 ± 0.1 a	68.3 ± 0.1 c	65 ± 1 f	64 ± 1 g	67 ± 2 d	67.7 ± 0. 4 c,d

Results are expressed mean ± SD (*n* = 3); dw—dry weight; SFA—Saturated fatty acids; MUFA—Monounsaturated fatty acids; PUFA—Polyunsaturated fatty acids. In each line, different letters indicate significant differences (*p* < 0.05) between samples.

**Table 3:** Antioxidant activity of hydromethanolic extracts of the studied *Agaricus subrufescens* samples produced in the field and in controlled environment crops.

		TBARS (EC_50_; mg/mL) *	OxHLIA (IC_50_; µg/mL, Δ*t* = 60 min) **
Field	AS 98/11	1.91 ± 0.05 a	221 ± 6 c
	AS 18/01	0.60 ± 0.04 h	88 ± 4 h,i
	AS CS7	0.44 ± 0.03 i	95 ± 3 g,h
	AS 19/01	1.89 ± 0.04 a	296 ± 9 b
	AS 16/01	0.50 ± 0.01 i	96 ± 2 g
	AS 04/49	0.93 ± 0.03 c,d	93 ± 4 g,h
Controlled Environment	AS 98/11	1.7 ± 0.1 b	310 ± 10 a
	AS 18/01	1.9 ± 0.1 a	111 ± 4 f
	AS CS7	0.96 ± 0.02 c	199 ± 1 d
	AS 19/01	0.72 ± 0.04 g	133 ± 5 e
	AS 16/01	0.88 ± 0.03 d,e	89 ± 3 g,h,i
	AS 04/49	0.8 ± 0.1 f	83 ± 2 j

Results are expressed mean ± SD (*n* = 3). In each line, different letters indicate significant differences (*p* < 0.05) between extracts. * EC_50_ values: extract concentration corresponding to 50% of antioxidant activity. ** IC_50_ values: extract concentration necessary to keep 50% of the erythrocyte population intact for 60 min. Trolox EC_50_ values: 0.023 ± 0.001 mg/mL (TBARS) and IC_50_ values: 21.8 ± 0.2 μg/mL (OxHLIA, Δ*t* 60 min).

**Table 4:** Antibacterial activity (MIC and MBC, mg/mL) of hydromethanolic extracts of the studied *Agaricus subrufescens* samples produced in the field and in controlled environment crops.

			Gram-Negative Bacteria					Gram-Positive Bacteria		
			*Escherichia* *coli*	*Klebsiella* *pneumoniae*	*Morganella morganii*	*Proteus* *mirabilis*	*Pseudomonas* *aeruginosa*	*Enterococcus* *faecalis*	*Listeria monocytogenes*	MRSA
Field	AS 98/11	MIC	20	>20	>20	>20	>20	>20	20	20
		MBC	>20	>20	>20	>20	>20	>20	>20	>20
	AS 18/01	MIC	20	>20	>20	>20	>20	>20	>20	20
		MBC	>20	>20	>20	>20	>20	>20	>20	>20
	AS CS7	MIC	20	>20	>20	>20	>20	20	20	20
		MBC	>20	>20	>20	>20	>20	>20	>20	>20
	AS 19/01	MIC	10	>20	>20	>20	>20	10	10	10
		MBC	>20	>20	>20	>20	>20	>20	>20	>20
	AS 16/01	MIC	20	>20	>20	>20	>20	20	20	20
		MBC	>20	>20	>20	>20	>20	>20	>20	>20
	AS 04/49	MIC	20	>20	>20	>20	>20	20	20	20
		MBC	>20	>20	>20	>20	>20	>20	>20	>20
Controlled environment	AS 98/11	MIC	20	>20	>20	>20	>20	>20	>20	20
		MBC	>20	>20	>20	>20	>20	>20	>20	>20
	AS 18/01	MIC	20	>20	>20	>20	>20	20	20	20
		MBC	>20	>20	>20	>20	>20	>20	>20	>20
	AS CS7	MIC	20	>20	>20	>20	>20	20	20	20
		MBC	>20	>20	>20	>20	>20	>20	>20	>20
	AS 19/01	MIC	10	>20	>20	>20	>20	10	10	10
		MBC	>20	>20	>20	>20	>20	>20	>20	>20
	AS 16/01	MIC	20	>20	>20	>20	>20	20	20	20
		MBC	>20	>20	>20	>20	>20	>20	>20	>20
	AS 04/49	MIC	20	>20	>20	>20	>20	20	20	20
		MBC	>20	>20	>20	>20	>20	>20	>20	>20
Negative controls	Ampicillin (20 mg/mL)	MIC	<0.15	10	>20	>20	>20	<0.15	<0.15	<0.15
		MBC	<0.15	20	>20	>20	>20	<0.15	<0.15	<0.15
	Imipenem (1 mg/mL)	MIC	<0.0078	<0.0078	<0.0078	<0.0078	0.5	n.t.	<0.0078	n.t.
		MBC	<0.0078	<0.0078	<0.0078	<0.0078	1	n.t.	<0.0078	n.t.
	Vancomycin (1 mg/mL)	MIC	n.t.	n.t.	n.t.	n.t.	n.t.	<0.0078	n.t.	0.25
		MBC	n.t.	n.t.	n.t.	n.t.	n.t.	<0.0078	n.t.	0.5

MRSA—Methicillin resistant *Staphylococcus aureus*; MIC—minimal inhibitory concentration; MBC—minimal bactericidal concentration; n.t.—not tested.

## Data Availability

All the data are mentioned in the manuscript and as supplementary materials.

## Supplementary Materials

The following are available online at https://www.mdpi.com/article/10.3390/jof8060616/s1, Table S1: Detailed fatty acid composition of *Agaricus subrufescens* samples produced in the field and in controlled environment crops. Table S2. Detailed tocopherols, phenolic acids and related compounds composition of *Agaricus subrufescens* samples produced in the field and in controlled environment crops.
