# Supplementary material for: Biochemical Approaches on Commercial Strains of Agaricus subrufescens Growing under Two Environmental Cultivation Conditions
Source: J Fungi (Basel). 2022 Jun 9;8(6):616. doi: 10.3390/jof8060616 (PMC9224743; doi:10.3390/jof8060616)
Supplement: Supplementary file 1 [file jof-08-00616-s001.zip › jof-1750690-supplementary.pdf]

Supplementary material

Biochemical approaches on Commercial Strains of *Agaricus subrufescens* Growing under two Environmental Cultivation Conditions

Daiana Almeida, Rossana V. C. Cardoso, Carla Pereira, Maria José Alves, Isabel C. F. R. Ferreira, Diego Cunha Zied, Wagner G. Vieira Junior, Cinthia E. C. Caitano, Ângela Fernandes, Lillian Barros

Table S1. Detailed fatty acid composition of *Agaricus subrufescens* samples produced in the field and in controlled environment crops.

	Field						Controlled environment					
	**AS 98/11**	**AS 18/01**	**AS CS7**	**AS 19/01**	**AS 16/01**	**AS 04/49**	**AS 98/11**	**AS 18/01**	**AS CS7**	**AS 19/01**	**AS 16/01**	**AS 04/49**
	**Fatty acids (%)**											
C6:0	0.187±0.004	0.15±0.01	1.6±0.1	0.50±0.01	0.180±0.005	0.21±0.01	0.190±0.004	0.26±0.02	0.57±0.01	0.40±0.04	0.37±0.01	0.29±0.02
C14:0	0.32±0.02	0.42±0.01	0.8±0.1	0.5±0.1	0.31±0.02	0.25±0.02	0.29±0.01	0.41±0.01	0.44±0.01	0.42±0.03	0.25±0.02	0.36±0.01
C15:0	0.9±0.1	0.94±0.05	1.75±0.02	1.1±0.1	0.75±0.04	0.828±0.001	0.85±0.02	0.93±0.01	1.02±0.01	1.1±0.1	0.92±0.05	0.9±0.1
C16:0	12.7±0.2	15±1	14.3±0.1	17.6±0.03	13.5±0.1	13.4±0.3	12.31±0.01	15.0±0.01	17±1	16.5±0.2	15±1	14±1
C16:1	0.173±0.005	0.25±0.02	0.35±0.01	0.37±0.01	0.32±0.03	0.19±0.01	0.108±0.005	0.18±0.02	0.157±0.001	0.11±0.01	0.13±0.01	0.17±0.01
C17:0	0.73±0.01	0.93±0.04	0.98±0.01	1.0±0.1	0.98±0.02	0.81±0.01	0.77±0.01	1.12±0.03	1.21±0.04	1.0±0.1	1.37±0.02	1.07±0.04
C18:0	3.8±0.1	4.9±0.3	4.42±0.07	4.97±0.02	5.0±0.1	4.4±0.2	4.250±0.001	4.6±0.3	5.23±0.01	5.15±0.05	4.9±0.3	4.9±0.3
C18:1n9c	1.2±0.1	2.1±0.2	1.54±0.03	1.5±0.1	1.6±0.1	1.28±0.03	1.19±0.03	1.5±0.1	1.9±0.1	2.0±0.1	1.6±0.1	1.4±0.1
C18:2n6c	73.44±0.16	68±1	66.2±0.2	64±1	70.0±0.3	71±1	72.8±0.1	68.1±0.1	64±1	64±1	67±2	67.5±0.4
C20:0	1.48±0.01	1.69±0.03	1.38±0.01	1.69±0.01	1.7±0.1	1.7±0.1	1.50±0.01	1.34±0.04	1.83±0.03	1.7±0.2	1.6±0.1	1.6±0.1
C20:1	0.33±0.02	0.38±0.04	0.55±0.01	0.35±0.02	0.23±0.01	0.38±0.04	0.32±0.01	0.32±0.03	0.14±0.01	0.474±0.004	0.37±0.01	0.37±0.03
C20:2	0.15±0.01	0.16±0.01	0.16±0.01	0.16±0.01	0.14±0.01	0.18±0.02	0.19±0.01	0.17±0.02	0.155±0.001	0.20±0.01	0.13±0.01	0.20±0.01
C21:0	0.37±0.02	0.41±0.01	0.78±0.02	0.45±0.04	0.34±0.03	0.42±0.02	0.39±0.01	0.43±0.03	0.55±0.01	0.44±0.02	0.46±0.03	0.49±0.02
C22:0	2.83±0.03	3.1±0.1	3.06±0.04	3.1±0.1	3.08±0.05	3.04±0.02	3.19±0.03	3.3±0.1	3.3±0.1	3.5±0.3	3.06±0.03	3.5±0.3
C23:0	0.54±0.03	0.66±0.01	1.04±0.03	1.4±0.1	0.7±0.02	1.29±0.04	0.55±0.02	1.1±0.1	1.2±0.1	1.4±0.1	1.3±0.1	1.42±0.04
C24:0	0.89±0.01	1.3±0.1	1.07±0.03	0.8±0.1	1.18±0.02	1.1±0.1	1.05±0.01	1.22±0.03	1.18±0.03	1.4±0.1	1.2±0.1	1.5±0.1
SFA	24.7±0.3	29±1	31.2±0.2	33±1	27.8±0.2	27±1	25.3±0.1	29.7±0.2	33±1	33±1	30±2	30.4±0.4
MUFA	1.7±0.1	2.8±0.2	2.44±0.01	2.2±0.1	2.1±0.1	1.8±0.1	1.62±0.04	2.0±0.2	2.2±0.1	2.6±0.1	2.1±0.1	1.94±0.03
PUFA	73.6±0.2	68±1	66.3±0.2	65±1	70.1±0.3	71±1	73.0±0.1	68.3±0.1	65±1	64±1	67±2	67.7±0.4

SFA - Saturated fatty acids; MUFA - Monounsaturated fatty acids; PUFA - Polyunsaturated fatty acids.

Table S2. Detailed tocopherols, phenolic acids and related compounds composition of *Agaricus subrufescens* samples produced in the field and in controlled environment crops.

	Field						Controlled environment					
	**AS 98/11**	**AS 18/01**	**AS CS7**	**AS 19/01**	**AS 16/01**	**AS 04/49**	**AS 98/11**	**AS 18/01**	**AS CS7**	**AS 19/01**	**AS 16/01**	**AS 04/49**
	Tocopherols (µg/100 g dw)											
α-Tocoferol	26.8±0.2c	20.5±0.6e	13.91±0.03i	16.7±0.1g	26.7±0.4c	47.5±0.6a	23.5±0.1d	17.1±0.7f	13.2±0.2j	14.5±0.7h	14.4±0.3h	40.4±0.3b
β-Tocoferol	76.0±0.3a	64.7±0.3b	57.6±0.1e	64.5±0.3b	60±1d	76.5±0.3a	65.0±0.6b	56.8±0.8f	50.72±0.03g	59.8±0.8d	36.6±0.1h	63±1c
δ-Tocoferol	44.1±0.6d	54.6±0.3c	35.5±0.2h	31.4±0.1i	42.7±0.8e	74.5±0.3a	36±1g	43.7±0.7d	20.6±0.1k	24.5±0.1j	40.3±0.1f	62.2±0.1b
Sum	146.9±0.1c	140±1d	107.0±0.1i	112.6±0.1h	130±1e	198.6±0.6a	124.6±0.6f	117.6±0.6g	84.4±0.4l	98.8±0.3j	91.2±0.4k	166±1b
	Phenolic acids and related compounds (µg/g dw)											
Protocatechuic acid	16.43±0.03e	11.62±0.01k	20.1±0.1a	19.73±0.08b	15.6±0.1g	15.9±0.1f	13.4±0.5j	11.2±0.2l	17.41±0.06d	18.5±0.2c	14.39±0.06h	13.89±0.04i
p-hydroxybenzoic acid	235.5±0.7b	189.1±0.7d	150.8±0.4j	194.3±0.4c	171.8±0.4g	167.0±0.2i	234±1a	184.3±0.2e	140±1k	173.71±0.08f	168.1±0.8h	138.7±0.4l
p-coumaric acid	69.5±0.7f	64.6±0.1i	74.3±0.5e	136.8±0.6c	171.7±0.8a	68.5±0.3g	57.6±0.1l	62.6±0.2j	66.61±0.06h	123.5±0.9d	162.8±0.3b	56.7±0.8k
Sum	321.4±0.1d	265.4±0.6g	245.2±0.1j	351±1b	359.0±0.5a	251.4±0.6i	305±1f	258.1±0.3h	224±1k	316±1e	345±1c	209.3±0.3l
Cinnamic acid	10.6±0.1j	16.6±0.2c	14.8±0.3e	14.08±0.03f	23.7±0.1a	13.12±0.07g	9.86±0.09k	15.66±0.04d	12.01±0.07h	13.2±0.1g	21.3±0.1b	11.1±0.1i

Tocopherols calibration curves: *α*-tocopherol ($y = 1.295x$; $R^2 = 0.991$; LOD = 18.06 ng/mL; LOQ = 60.20 ng/mL), β-tocopherol ($y = 0.396x$; $R^2 = 0.992$; LOD 25.82 ng/mL; LOQ = 86.07 ng/mL), and δ-tocopherol ($y = 0.678x$; $R^2 = 0.992$; LOD = 20.09 ng/mL; LOQ = 66.95 ng/mL). Phenolic acids and related compounds calibration curves: protocatechuic acid ($y = 166966x + 251225$; $R^2 = 0.9978$); *p* hydroxybenzoic acid ($y = 21.680x + 9264.2$; $R^2 = 0.9983$); *p*-coumaric acid ($y = 93234x + 56858$; $R^2 = 0.9936$) and cinnamic acid ($y = 135559x + 222170$; $R^2 = 0.9955$).
